# Bilateral same-day endoscopic transcanal cartilage tympanoplasty: initial results^☆^

**DOI:** 10.1016/j.bjorl.2016.04.014

**Published:** 2016-05-24

**Authors:** Ahmad Daneshi, Hesam Jahandideh, Ali Daneshvar, Mahdi Safdarian

**Affiliations:** Iran University of Medical Sciences, Rasoul Akram Hospital, ENT and Head & Neck Surgery Research Center, Tehran, Iran

**Keywords:** Cartilage, Endoscopy, Same-day, Tympanic membrane perforation, Tympanoplasty, Cartilagem, Endoscopia, Mesmo dia, Perfuração da membrana timpânica, Timpanoplastia

## Abstract

**Introduction:**

Same-day closure of bilateral tympanic membrane perforations is a quick and more comfortable procedure for the patients. However, conventional bilateral same-day tympanoplasty or myringoplasty has been rarely performed because of the theoretical risk of postoperative complications.

**Objective:**

To evaluate the advantages and outcomes of bilateral simultaneous endoscopic cartilage tympanoplasty in patients with bilateral tympanic membrane perforations.

**Methods:**

From February 2012 to March 2013, patients with bilateral dry tympanic membrane perforations who had some degree of hearing loss corresponding to the size and location of the perforation entered the study. There was no suspicion to disrupted ossicular chain, mastoid involvement or other middle or inner ear pathology. Endoscopic transcanal cartilage tympanoplasty was done using the underlay (medial) technique. The graft was harvested from cymba cartilage in just one ear with preservation of perichondrium in one side. A 1.5 cm × 1.5 cm cartilage seemed to be enough for tympanoplasty in both sides.

**Results:**

Nine patients (4 males and 5 females) with the mean age of 37.9 years underwent bilateral transcanal cartilage tympanoplasty in a same-day surgery. The mean duration of follow up was 15.8 months. There were detected no complications including hearing loss, otorrhea and wound complication with no retraction pocket or displaced graft during follow-up period. The grafts take rate was 94.44% (only one case of unilateral incomplete closure). The mean of air-bone gap overall improved from 13.88 dB preoperatively to 9.16 dB postoperatively (*p* < 0.05).

**Conclusion:**

Bilateral endoscopic transcanal cartilage tympanoplasty can be considered as a safe minimally invasive procedure that can be performed in a same-day surgery. It reduces the costs and operation time and is practical with a low rate of postoperative complications.

## Resumo

**Introdução:**

O fechamento no mesmo dia de perfuração bilateral da membrana timpânica é um procedimento rápido e mais confortável para os pacientes. Entretanto, a timpanoplastia ou miringoplastia convencional bilateral executada no mesmo procedimento tem sido raramente realizada devido ao risco teórico de complicações pós-operatórias.

**Objetivo:**

Avaliar as vantagens e resultados da timpanoplastia bilateral simultânea com cartilagem por via endoscópica em pacientes com perfuração bilateral da membrana timpânica.

**Método:**

De fevereiro de 2012 a março de 2013, pacientes com perfuração seca bilateral da membrana timpânica que tinham algum grau de perda de audição correspondente ao tamanho e localização da perfuração foram incluídos no estudo. Não houve suspeita de cadeia ossicular interrompida, envolvimento do mastoide ou outra doença da orelha média ou interna. Timpanoplastia com cartilagem transcanal foi executada por via endoscópica usando a técnica de *underlay* (medial). O enxerto foi colhido de cartilagem da concha superior (cymba) em apenas uma orelha com preservação do pericôndrio em um lado. Um enxerto de 1,5 × 1,5 centímetros de cartilagem pareceu ser o suficiente para a timpanoplastia em ambos os lados.

**Resultados:**

Nove pacientes (4 homens e 5 mulheres) com idade média de 37,9 anos foram submetidos à timpanoplastia bilateral com cartilagem transcanal em uma cirurgia realizada em etapa única. O tempo médio de acompanhamento foi de 15,8 meses. Não foram detectadas complicações, incluindo perda de audição, otorreia e complicações como bolsa de retração ou deslocamento de enxerto durante o período de seguimento. A taxa de sucesso do enxerto foi de 94,44% (apenas um caso de fechamento unilateral incompleto). A média do gap aéreo-ósseo em geral melhorou de 13,88 dB no pré-operatório para 9,16 dB no pós-operatório (*p* < 0,05).

**Conclusão:**

Timpanoplastia bilateral com cartilagem transcanal por via endoscópica pode ser considerada como um procedimento minimamente invasivo, seguro e que pode ser executado em uma única cirurgia. Isso reduz os custos e tempo de operação e é prático, com um baixo índice de complicações pós-operatórias.

## Introduction

Tympanoplasty is the standard and well-established procedure for closure of tympanic membrane perforations. Traditionally each eardrum was taken up for grafting sequentially in two different settings, which leads to considerable increase in operation cost, time and discomfort to the patient.^1^

Same-day closure of bilateral tympanic membrane perforations is a quick and more comfortable procedure for the patients. However, conventional bilateral same-day tympanoplasty or myringoplasty has been rarely performed because of the theoretical risk of postoperative complications.^2^ The risk of iatrogenic hearing loss related to bilateral tympanoplasty on the same day is reported to be about 1.2–4.5%. Therefore, the conventional belief amongst otosurgeons is to avoid doing bilateral tympanoplasty simultaneously.^3^, ^4^, ^5^

In this study, we evaluated the advantages and outcomes of performing bilateral simultaneous endoscopic cartilage tympanoplasty in a case series of patients with bilateral tympanic membrane perforations.

## Methods

Institutional ethical clearance obtained prior to conduction of this study from the local ethics committee of ENT-Head & Neck surgery research center of Rasoul Akram Hospital, Iran University of Medical Sciences Tehran, Iran and the patients has consented for submission of this paper to the journal.

Nine patients with bilateral dry tympanic membrane perforations entered the study. All patients had some degree of hearing loss (all of them less than 30 dB) that seemed corresponding to the size and location of the perforation. There was no suspicion to disrupted ossicular chain, mastoid involvement or other middle or inner ear pathology. All the patients had at least 2 months dry ear prior to the surgery, which is logically suitable for performing a regular tympanoplasty.

The patients were placed in the supine position, with head 30 degrees up and turned slightly toward the side to be operated. The video equipment was placed in front of the surgeon and the instrument trolley and scrub nurse were positioned at the head. We used Karl Storz (Tuttlingen, Germany) high definition monitor and camera and 4 mm in diameter and 18 cm long endoscopes with 0 and 30 degrees of angulation were used. Endoscopic transcanal cartilage tympanoplasty was done using the underlay (medial) technique. The graft was harvested from cymba cartilage in just one ear with preservation of perichondrium in one side. A 1.5 cm × 1.5 cm cartilage seemed to be enough for tympanoplasty in both sides.

**Figure 1:**
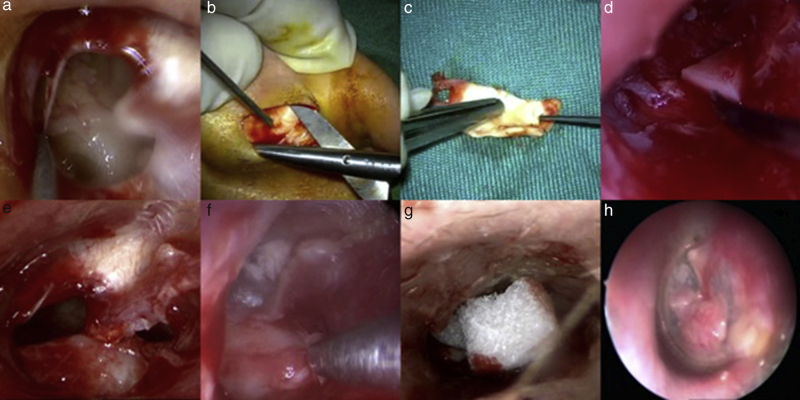
Endoscopic transcanal tympanoplasty. At first, a minimal amount of tissue removed in order to get back to fresh edges (A); both grafts harvested from one side (B) and their shapes were designed based on the perforations (C). A wedge of cartilage was used over promontory to hold the cartilage (D); or in some cases, a small piece of cartilage was placed into the hypotympanum (E). The graft was placed with underlay technique while perichondrium layer facing laterally (F); for preventing displacement of the graft, small pieces of gelfoam were placed over cartilage into the canal (G). Post operative result (H).

In first step, a minimal amount of tissue removed in order to get a fresh edge (Fig. 1A). We did not need to elevate any tympanomeatal flap in our new technique. Then graft harvested from one ear (Fig. 1B) and its shape was designed based on the shape of perforation (Fig. 1C). A wedge of cartilage was used over promontory to hold the cartilage in order to prevent the blockage of the eustachian tube (Fig. 1D). In cases that needed greater support for graft, small piece of cartilage was placed into the hypotympanum (Fig. 1E) or if needed it was placed superiorly, just as described by Tos.^6^ Then graft was placed with underlay technique while perichondrium layer facing laterally (Fig. 1F). For preventing displacement of graft, small pieces of gelfoam placed over cartilage into the canal (Fig. 1H).

Paired *t*-test was used to compare the mean of air-bone gap before and after operation using SPSS software version 20 (SPSS Inc, Chicago, IL, USA). *p*-Value less than 0.05 was considered as significantly meaningful.

## Results

From February 2012 to March 2013, 9 patients (4 males and 5 females) underwent bilateral transcanal cartilage tympanoplasty in a same-day surgery by the first author (AD) under general or local anesthesia. The mean age of patients was 37.9 years and the mean duration of follow up was 15.8 months (Table 1). Follow-up examination and hearing tests (pure tone audiometry) were performed up to 20 months after surgery. During the follow up period, there were no complications including hearing loss, otorrhea and wound complication.

**Table 1:** Patients’ data.

Number	Gender	Age (year)	Duration of follow-up (month)	Anatomical location of the perforation		Preoperative ABG (dB)		Postoperative ABG (dB)	
				Right ear	Left ear	Right ear	Left ear	Right ear	Left ear
1	F	40	12	Central	Central	15	20	15	10
2	F	58	20	Posterior	Central	10	15	10	10
3	F	12	16	Central	Anterior	15	10	10	5
4	M	60	16	Anterior	Central	15	10	5	5
5	F	33	13	Posterior	Posterior	10	10	5	10
6	F	45	15	Central	Anterior	20	15	15	10
7	M	35	18	Central	Posterior	15	15	10	5
8	M	30	15	Central	Central	20	15	10	10
9	M	28	17	Central	Anterior	10	10	10	5

ABG, air-bone gap.

No retraction pocket or displaced graft was observed during follow-up. The grafts take rate was 94.44% (only one case of unilateral incomplete closure). The patient number six with the perforation in the anterior part of right tympanic membrane had an unclosed perforation, which was repaired later with lobule fat under local anesthesia. The mean of air-bone gap overall improved from 13.88 dB preoperatively to 9.16 dB postoperatively (*p* < 0.05).

The first post-operative audiometric exam performed 2 months after surgery and then every 6 months. To the time of this report, all the postoperative audiometric exams were unremarkable and we had no otorrhea postoperative.

## Discussion

Endoscopic approach to the middle ear and tympanic cavity is a practical, minimally invasive and conservative technique in comparison to the traditional surgical approaches. In this method, there will be no more need to use several flaps or performing canaloplasty. As a result, there will be no disturbance in the external ear blood circulation.

Postoperative ear packing in conventional surgeries, which leads to considerable hearing loss and causes a transient activity limitation to the patient, is not applied in endoscopic method since all the procedure is done with only few stiches in cartilage harvesting site without any other postauricular incisions.^7^

Endoscopic tympanoplasty is time saving and anatomy of the middle ear will be preserved. This procedure does not require surgical exposure such as canal drilling and skin incision, and avoids the substantial risk of unnecessary injury to the chorda tympani, in contrast to conventional methods.

Less pain, reduced demand for analgesics and reduced time of operation and a shorter period of follow-up are the other advantages of the endoscopic method. Something that makes this procedure unique is the possibility of performing bilateral tympanoplasty at the same time without a necessarily general anesthesia. Three of our cases in this study received sedation and the other six patients received general anesthesia. In the cases of the selected patients with underlying medical problems or patients’ own preference, intravenous sedation can be done instead of general anesthesia.

Endoscopic tympanoplasty is possible to be performed with 0 or 30 degree telescopes with no need to any bone drilling. Since microscope is not used during the procedure and it is possible to see the ossicular chain and the middle ear through a 30 degree telescope. This method is particular useful in cases of anterior canal overhang without need to remove the overhang.

Marchioni et al. showed better results for endoscopic transcanal approach to the tympanic cavity for management of cholesteatoma in pediatric patients in comparison to the group of canal wall up microscopic approach.^8^

In the study by Dundar et al., comparing endoscopic and microscopic tympanoplasty in 60 children, the endoscopic and microscopic approaches were reported to give equal results in terms of easy visualization of the entire tympanic membrane and requirement for extra intervention to evaluate the ossicular system. Nevertheless, a shorter operative duration was mentioned to be an advantage of the endoscopic tympanoplasty technique.^9^

Kakehata et al. followed nine patients who underwent endoscopic transtympanic tympanoplasty for an average period of 17 months to report their experience in the treatment of conductive hearing loss. The patients showed an average improved hearing level of 32 dB with an average air-bone gap of 11 dB. They recommended endoscopic transtympanic tympanoplasty as an adequate and minimally invasive procedure for a disrupted ossicular chain.^10^

A theoretical risk of iatrogenic sensorineural hearing loss during surgery has induced a reluctance to perform bilateral tympanoplasty among some otosurgeons,^11^ while many studies represent bilateral same-day tympanoplasty as a feasible treatment option in the middle ear pathologies such as chronic (suppurative) otitis media.^1^, ^11^, ^12^, ^13^

Moreover, even in stapes surgery with greater theoretical risk for hearing loss, we had no case of dead ear, facial nerve paralysis or intraoperative chorda tympani nerve transection in our previous series of endoscopic procedures.^14^

Kim et al. showed that bilateral simultaneous middle ear surgery provides good hearing outcomes, reduces costs and operation times, and has a low incidence of complications.^15^

To avoid complications especially to the ossicular chain, we tried to include patients with a narrow preoperative GAP. Considering different kinds of perforations in this study, we tried to keep the proper distance with ottic in order to avoid injury to the middle ear components. We also tried to provide a good visual field on ossicular chain during the operation using different angled endoscopes in addition to utilizing precise surgery technique. We also avoided the overheating of middle ear components by frequent repositioning and keeping the endoscope in a proper distance to allow tissue cooling.^16^

In order to maintain the graft in position, we used pieces of cartilage in contact with the middle ear mucosa. In order to avoid inducing fibrosis and reducing the tympanic membrane sound absorption and transmission, the cartilage was used as a support placing on the promontory and in the hypotympanum cavity in order not to have any interference with the middle ear function.

In our study, a significant improvement was seen in the mean of air-bone gap while there were no complications during the follow up period. Only one case of unilateral incomplete closure was detected and all the other eight grafts were taken successfully.

## Conclusion

Bilateral endoscopic transcanal cartilage tympanoplasty is a safe minimally invasive procedure that can be performed in a same-day surgery. It reduces the costs and operation time and is practical with a low rate of postoperative complications.

## Conflicts of interest

The authors declare no conflicts of interest.
